# Supplementary material for: Resolving the full spectrum of human genome variation using Linked-Reads
Source: Genome Res. 2019 Apr;29(4):635–45. doi: 10.1101/gr.234443.118 (PMC6442396; doi:10.1101/gr.234443.118)
Supplement: Supplemental Material [file supp_gr.234443.118_Supplemental_longranger-2.2.zip › longranger-2.2.tar.bz2!/lr2.2-public/lib/stan/cmdstan/stan/doc/api/html/arg__test__grad__err_8hpp_source.html]

Stan: src/stan/gm/arguments/arg\_test\_grad\_err.hpp Source File


|  |  |
| --- | --- |
|  | Stan  2.5.0  probability, sampling & optimization |


- Main Page
- Related Pages
- Namespaces
- Classes
- Files

- File List
- File Members

All Classes Namespaces Files Functions Variables Typedefs Enumerations Enumerator Friends Macros Pages

- src
- stan
- gm
- arguments

arg\_test\_grad\_err.hpp

Go to the documentation of this file.

1 #ifndef STAN\_\_GM\_\_ARGUMENTS\_\_TEST\_\_GRAD\_\_ERR\_\_HPP

2 #define STAN\_\_GM\_\_ARGUMENTS\_\_TEST\_\_GRAD\_\_ERR\_\_HPP

3

4 #include <stan/gm/arguments/singleton\_argument.hpp>

5

6 namespace stan {

7

8  namespace gm {

9

10  class arg\_test\_grad\_err: public real\_argument {

11

12  public:

13

14  arg\_test\_grad\_err(): real\_argument() {

15  \_name = "error";

16  \_description = "Error threshold";

17  \_validity = "0 < error";

18  \_default = "1e-6";

19  \_default\_value = 1e-6;

20  \_constrained = true;

21  \_good\_value = 1e-6;

22  \_bad\_value = -1.0;

23  \_value = \_default\_value;

24  };

25

26  bool is\_valid(double value) { return value > 0; }

27

28  };

29

30  } // gm

31

32 } // stan

33

34 #endif

stan::gm::singleton\_argument::\_validity

std::string \_validity

**Definition:** singleton\_argument.hpp:151

stan::gm::arg\_test\_grad\_err::is\_valid

bool is\_valid(double value)

**Definition:** arg\_test\_grad\_err.hpp:26

stan::gm::singleton\_argument::\_value

T \_value

**Definition:** singleton\_argument.hpp:154

stan::gm::valued\_argument::\_default

std::string \_default

**Definition:** valued\_argument.hpp:47

stan::gm::singleton\_argument

**Definition:** singleton\_argument.hpp:44

stan::gm::singleton\_argument::\_bad\_value

T \_bad\_value

**Definition:** singleton\_argument.hpp:160

stan::gm::singleton\_argument::value

T value()

**Definition:** singleton\_argument.hpp:124

stan::gm::argument::\_name

std::string \_name

**Definition:** argument.hpp:76

stan::gm::singleton\_argument::\_constrained

bool \_constrained

**Definition:** singleton\_argument.hpp:157

stan::gm::arg\_test\_grad\_err

**Definition:** arg\_test\_grad\_err.hpp:10

stan::math::e

double e()

Return the base of the natural logarithm.

**Definition:** constants.hpp:86

stan::gm::arg\_test\_grad\_err::arg\_test\_grad\_err

arg\_test\_grad\_err()

**Definition:** arg\_test\_grad\_err.hpp:14

stan::gm::singleton\_argument::\_default\_value

T \_default\_value

**Definition:** singleton\_argument.hpp:155

singleton\_argument.hpp

stan::gm::singleton\_argument::\_good\_value

T \_good\_value

**Definition:** singleton\_argument.hpp:159

stan::gm::argument::\_description

std::string \_description

**Definition:** argument.hpp:77


---

[ Stan Home Page ]

*© 2011–2014,
Stan Development Team.*
